# M. Monnot, on the Membranous Cataract

**Published:** 1804-01-01

**Authors:** R. Hall

**Affiliations:** Clement's Inn


### To the Editors of the Medical and Physical Journal.

Gentlemen,

In Duncan's Annals for 1802, I observe a paper, by M. Monnot, Professor of Anatomy and Midwifery, at Besançon, containing in my opinion, some useful remarks on what is termed the Membranous Cataract, or that obfuscation consequent on the inflammation of the capsule, after the removal of the crystalline lens. As, however, the utility of these observations must be extremely confined from being presented to the public, by the editors of that work, in the original French, if you deem the following short account of them worthy of insertion in your useful Journal, it is much at your service.

*Clement's Inn,* I am, &c.

*December* 14, 1803. R. HALL.

---

Practitioners have, it is well known, been long divided in opinion as to the respective merits of the two operations of couching, and extracting the cataract. In this paper M. Monnot does not however enter into any discussion on this question, but merely confines himself to the improvement of the latter operation. He commences with a brief history of cataract, which, he observes, is first mentioned in the writings of Hippocrates, who describes with great accuracy the appearances of this malady, although he seems to have been wholly unacquainted with its true character, as well as the operation necessary for its removal; circumstances solely attributable to the impossibility, at that period, of ascertaining by anatomical examination the nature of the body intercepting the rays of light.

From that period until the time of Celsus, this department of surgery appears to have made very inconsiderable progress, as this Latin author was the first who recommended any operation for the cure of cataract.

It would be here unnecessary to follow the author in his detail of the different opinions maintained by various writers, respecting the nature and seat of this disease; according to him, the certainty that it proceeds from an opacity of the crystalline lens, was first demonstrated by Antoine Maitre Jean, but the method of operating practised by Celsus was nevertheless followed, with little variation, until the commencement of the last century.

The

The first example of the lens being displaced from the posterior chamber of the eye, by means of a needle passed through the pupil, is recorded by De Mery, in the Memoirs of the Academy of Sciences for 1707; since which period various improvements have been made in the manner of conducting this operation, and on the instruments necessary in performing it.

M. Monnot divides cataract into true and false; the first, according to our author, proceeds from the lens acquiring a degree of opacity, which prevents the rays of light falling on the retina; the second, is owing to a total or partial dissolution of the crystalline, within its capsule, which he denominates the white or milky cataract; whilst the want of transparency in the capsule itself, is termed by him the membranous cataract. This last affection, which frequently succeeds the extraction of the crystalline lens, deprives the patient of sight, however dexterously the operation may be performed.

" This occurrence," observes M. Monnot, " may be prevented, if an intelligent and steady operator divide the posterior part of the capsule, which separates, and allows the vitreous humour to occupy the place of the crystalline lens. This membrane, being removed from the focus through which the light is directed, no longer impedes its access.

" To explain the superior advantages of this method over that commonly employed, I shall," continues the author, " particularly explain the mode of its performance.

" The patient being placed near a window, and the operator before him on a more elevated seat, his head rests on the breast of an assistant, who fixes it, by placing one hand on the forehead, whilst, with the other, he raises the upper eyelid. In order to prevent the motion of the organ, the other eye is covered with some compresses, supported by a bandage. The operator draws down the lower lid, without pressing on the organ, and takes, with the other hand, the bistoury from an assistant. He directs the point to the edge of the transparent cornea, and to the middle part, which answers to the lesser angle of the eye. Instead of directing it transversely, from the little to the great angle, he conducts it obliquely, so that the point of the instrument is in a straight line with the angle of the nose. This direction, which should be that of every cutting instrument, renders the wound more regular, and is less difficult in the execution.

"When the wound is made on the transparent cornea, and the eye gently pressed, the lens breaks the slight connexion which retained it, and falls on the cheek. Where the capsule resists, a slender instrument, terminated like a serpent's tongue, passed by the pupil, and carried over the anterior part of the crystalline lens, divides it; and this body escapes by the opening."

After this part of the operation is finished, nothing more is supposed necessary by most operators; but, as the author has observed, blindness frequently occurs, in consequence of the inflammation of the capsule of the lens, constituting the affection styled the membranous cataract, he advises, in order to prevent this distressing accident, that immediately after the extraction of the lens, the same instrument should be directed to the posterior part of the capsule, with which a crucial incision should be made on that part. Immediately the vitreous humour occupies the place of the crystalline lens, and the eye remains clear, without a cloud.

Among other examples of the success of this method, M. Monnot relates the case of a domestic of M. Chalon, at Besançon, above sixty years of age, affected with a cataract in both eyes. On one of them he operated in the usual way; whilst, on the other, he likewise made an incision on the posterior part of the capsule. On finishing the operation, the patient recovered the sight in both eyes, but only retained it permanently in the eye in which that membrane had been divided.

By dissecting the eyes of patients who died blind, notwithstanding they had undergone the operation of extraction, the author discovered, that, in these cases, the privation of sight was solely imputable to a morbid thickening in the posterior part of the capsule; an effect which, he ventures to affirm, may be almost always avoided, by having recourse to the operative process here recommended.

*Extract*

